# Pathological complete response to neoadjuvant therapy with serplulimab and chemotherapy in stage IIIB small cell lung cancer: a case report and literature review

**DOI:** 10.3389/fimmu.2023.1272450

**Published:** 2024-01-18

**Authors:** Ting Mei, Ting Wang, Chuanfen Lei, Dan Jiang, Qinghua Zhou

**Affiliations:** ^1^ Department of Medical Oncology, Cancer Center, West China Hospital, Sichuan University, Chengdu, Sichuan, China; ^2^ Lung Cancer Center, West China Hospital, Sichuan University, Chengdu, Sichuan, China; ^3^ Department of Pathology, West China Hospital, Sichuan University, Chengdu, Sichuan, China

**Keywords:** small cell lung cancer, immunotherapy, neoadjuvant therapy, pathological complete response, serplulimab, case report

## Abstract

Chemotherapy combined with immunotherapy has significantly improved survival in patients with extensive-stage small cell lung cancer (ES-SCLC), and neoadjuvant immunotherapy combined with chemotherapy has emerged as the standard treatment for those with resectable non-small cell lung cancer (NSCLC). However, the potential benefits of surgery following neoadjuvant immunotherapy combined with chemotherapy in locally advanced SCLC remain unclear. Herein, we report a patient diagnosed with stage IIIB SCLC, who was administered five cycles of neoadjuvant serplulimab combined with chemotherapy followed by surgery, and subsequently achieved a pathologic complete response (pCR). Within a follow-up duration of six months, the patient displayed neither recurrence nor metastasis and experienced no treatment-related adverse reactions of any grade. Based on this case, for locally advanced SCLC, neoadjuvant serplulimab combined with chemotherapy followed by surgery may present an effective, safe, and potentially curative treatment strategy. Nonetheless, further prospective studies are needed to verify our findings.

## Introduction

Small cell lung cancer (SCLC) accounts for approximately 13-15% of all lung cancer cases (1). Characterized by its rapid progression, aggressive behavior, and propensity for distant metastasis, SCLC is often associated with a poor prognosis (2). Approximately 1/3 of SCLC patients are in the limited stage (LS-SCLC) at the time of initial diagnosis (3). Although LS-SCLC is highly sensitive to chemotherapy and radiotherapy, the median survival is limited to 16-24 months, emphasizing the urgent need to improve efficacy and expand the scope of current treatment strategies (4).

Surgery, an integral facet of multimodal cancer management, is currently only recommended for patients with T1-2N0M0 SCLC (5). And a study has shown that stage IIIA SCLC patients who receive neoadjuvant chemotherapy have a disappointingly low postoperative pCR rate of only 5% (6). Given these considerations, the role of surgery in managing stage III SCLC remains contentious, and their standard treatment regimen is concurrent chemoradiotherapy.

Results from the Checkmate 816 study demonstrated that neoadjuvant immunotherapy combined with chemotherapy extended event-free survival (EFS) and elevated rates of pCR compared with chemotherapy alone in patients with resectable NSCLC (7). A previous case report by Zhang et al. also showed that neoadjuvant tislelizumab combined with chemotherapy brought LS-SCLC patients an event-free survival of up to 23 months (8). Moreover, previous IMpower133 and CASPIAN studies have established that incorporating PD-L1 inhibitors into chemotherapy can significantly improve the overall survival (OS) of patients with ES-SCLC (9, 10). Serplulimab, a PD-1 inhibitor characterized by its strong affinity and low immunogenicity, has been substantiated in the ASTRUM-005 study to effectively improve the prognosis of ES-SCLC patients when combined with chemotherapy, yielding a median progression-free survival (PFS) and OS of 5.7 and 15.4 months, respectively (11). Based on these observations, we hypothesized that after LS-SCLC received neoadjuvant immunotherapy combined with chemotherapy, the tumor might undergo further shrinkage and down-staging, thus allowing for more comprehensive resection and potentially resulting in enhanced survival. However, there are very few data on stage III SCLC patients who received neoadjuvant chemotherapy combined with immunotherapy followed by surgery.

Here we report a case of stage IIIB SCLC patient who underwent five cycles of neoadjuvant serplulimab combined with chemotherapy and achieved pCR after surgery.

## Case report

### General conditions

**Figure 1:**
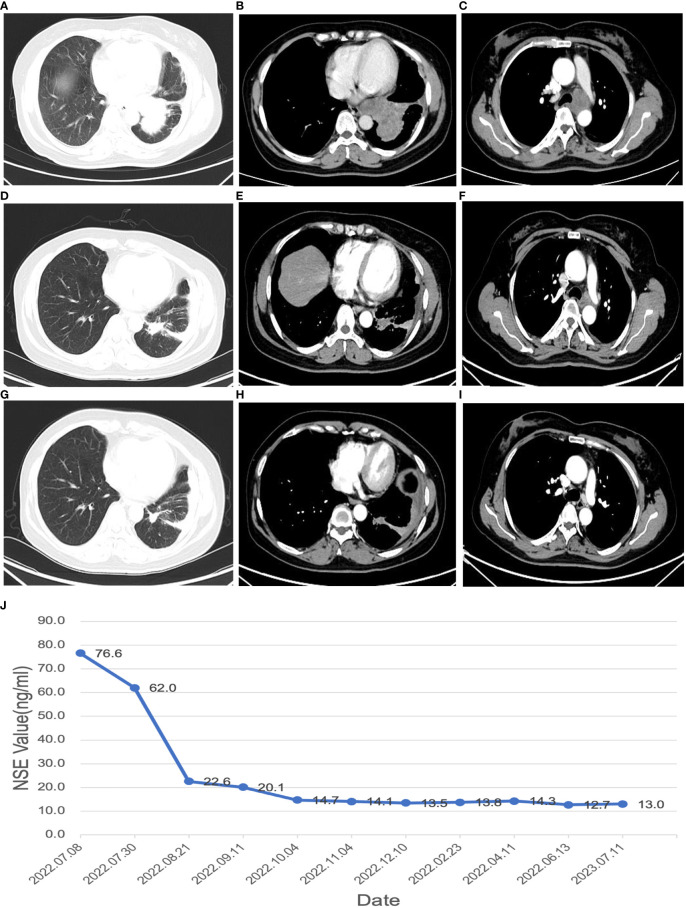
Changes in maximum tumor volume by chest CT scans and changes in NSE value during the disease **(A–C)**, CT images at the time of initial diagnosis; **(D–F)**, CT images after receiving 2 cycles of neoadjuvant therapy; **(G–I)**, CT images after 4 cycles of neoadjuvant therapy; **(J)**, changes in NSE value during the disease.

In July 2022, a 51-year-old female presented to the West China Hospital of Sichuan University with complaints of persistent cough and sputum production. She reported no history of smoking or any notable medical conditions. On physical examination, no palpable lymphadenopathy was detected throughout her body and her cardiopulmonary examination was unremarkable. Blood tests revealed elevated levels of enolase (76.6ng/ml). The contrast-enhanced chest computed tomography (CT) demonstrated a mass measuring approximately 9 cm x 6.9 cm in the lower lobe of the left lung, encasing a part of the left lower lobe bronchi and resulting in occlusion of the corresponding bronchial lumen (**Figures 1A, B**). Additionally, enlargement of mediastinal and left hilar lymph nodes was observed, with a maximum size of 3.4 cm x 3.2 cm (**Figure 1C**). Contrast-enhanced abdominal CT, brain magnetic resonance imaging (MRI), and bone scan all returned normal findings. A bronchoscopic biopsy conducted via a fiberoptic bronchoscope from the basal segment of the left lower lobe confirmed the diagnosis of SCLC. Immunohistochemical profile was as follows: CK7(−), TTF-1(+), NapsinA(−), CK5/6(−), P63(−), Syn(+), CK(Pan)(+), CD56(+), CgA(+), Ki-67(MIB-1,+, 90%), P53(+), RB(−,expression loss). EBER (*in situ* hybridization, −).

### Diagnosis and treatment process

The patient was diagnosed with SCLC of the left lower lobe with metastasis to the left hilar and mediastinal lymph nodes, classified as cT4N2M0 stage IIIB. Despite concurrent chemoradiotherapy being the standard treatment recommended by the guidelines for such cases, the patient expressed a strong preference for surgery. Upon consultation with a multidisciplinary team, it was considered that the patient’s tumor was potentially resectable. Based on the Checkmate 816 study, we posited that a preoperative regimen of neoadjuvant immunotherapy and chemotherapy followed by surgery might be an optimal strategy. Moreover, Zhang et al. also previously reported that stage IIIB SCLC patients obtained pCR after receiving neoadjuvant tislelizumab combined with chemotherapy, and the recurrence-free survival time was as long as 23 months. In addition, the results of the ASTRUM-005 study showed that serplulimab combined with chemotherapy significantly improved the OS of patients with ES-SCLC. The patient ultimately opted for a neoadjuvant regimen consisting of etoposide (100 mg/m^2^, days 1-3), cisplatin (75 mg/m^2^, days 1-3), and serplulimab (300 mg, day 1). She received five cycles of this combined EP with a serplulimab regimen, specifically: etoposide 150mg on days 1-3, cisplatin 40mg on days 1-2 and 30mg on day 3, and serplulimab 300mg on day 1, administered intravenously every three weeks.

## Results

**Figure 2:**
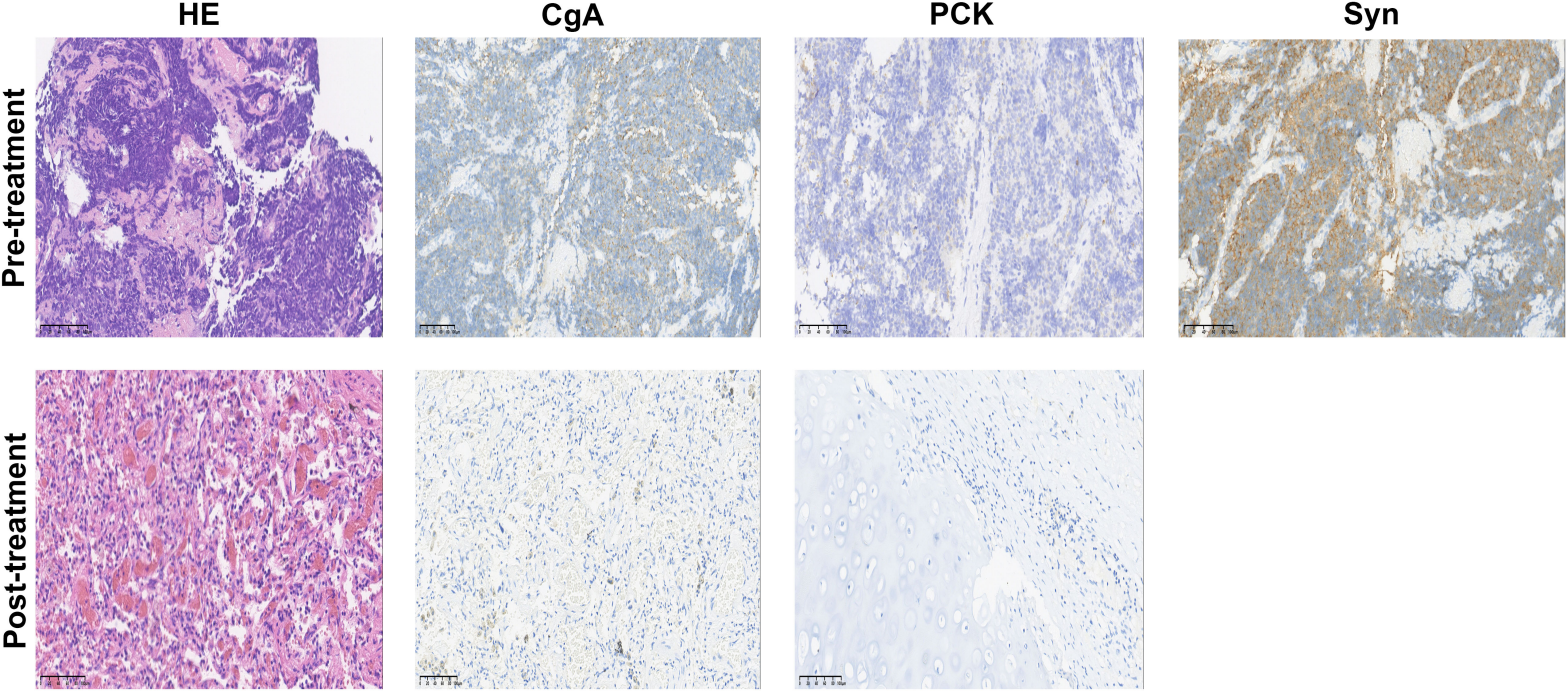
Hematoxylin-eosin (HE) and immunohistochemistry staining in the tumor bed. Staining of the post-treatment lung mass showed no viable tumor cells; pan-cytokeratin (PCK) and chromogranin A (CgA) were negative; Syn staining was omitted due to the absence of remaining tumor cells after treatment. Scale bar, 100 µm.

**Figure 3:**
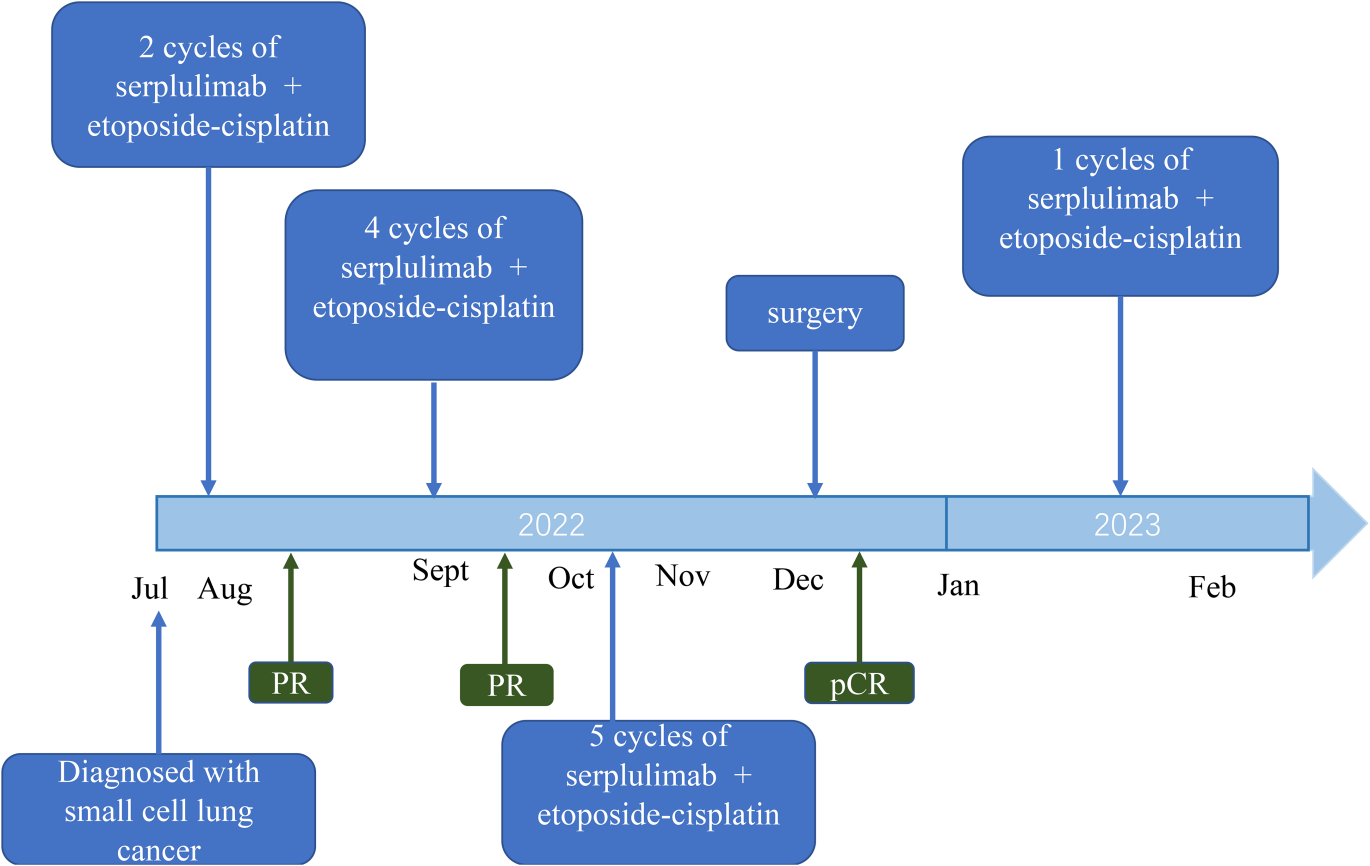
Timeline from July 2022 to July 2023.

Following two cycles of neoadjuvant therapy, the patient’s left lower lobe lesion diminished to 3.5 cm, and the size of the largest mediastinal lymph node reduced to 2.5 cm x 2.0 cm (**Figures 1D–F**), demonstrating a partial response (PR) to the treatment. After completing four cycles of neoadjuvant therapy, the left lower lobe lesion in the patient had further reduced to 1.5 cm, and the largest mediastinal lymph node had shrunken to 1.8 cm x 1.0 cm (**Figures 1G–I**), signifying a sustained PR to the treatment. After 5 cycles of treatment, the tumor did not shrink further, nor did the NSE level decrease further. The changes in NSE values during the course of the disease are shown in **Figure 1J**. And the patient underwent a left lower lobectomy coupled with lymph node dissection 47 days after the completion of the neoadjuvant therapy. Postoperatively, the treatment efficacy was evaluated as a pCR (**Figure 2**). Post-surgery, the patient was administered one cycle of adjuvant therapy combining EP regimen with serplulimab, and then did not receive serplulimab maintenance therapy. Routine imaging evaluations were conducted bimonthly during the follow-up period. From the time of receiving neoadjuvant serplulimab combined with chemotherapy to the cut-off time of follow-up, which lasted up to 12 months, no tumor recurrence or metastasis was found (**Figure 3**). No adverse effects were noted during treatment.

## Discussion

The current NCCN guidelines only recommend radical surgery for LS-SCLC patients with T1-2N0M0 staging (5). However, less than 5% of initially diagnosed SCLC patients meet this standard (12). For the majority of LS-SCLC patients, concurrent chemoradiotherapy is the standard treatment modality, despite the high response rate of up to 80% for first-line treatment, and most patients experience relapse within six months of completing their initial treatment (13). Therefore, the pursuit of more effective therapeutic strategies remains an ongoing focus of our research endeavors.

Historically, surgery has not been recommended treatment for SCLC, a position informed by the findings of two large-scale, randomized prospective trials. The MRC study, executed by the UK Medical Research Council in the 1960s, demonstrated a superiority of definitive radiotherapy over surgery in patients with LS-SCLC, evidenced by a notably longer OS (14, 15). Adding to this, another multicenter randomized phase III study by the Lung Cancer Study Group (LCSG) further supported the non-recommendation of surgery (16). In that study, 146 LS-SCLC patients with regional lymph nodes involvement (T3N1-2M0) received five cycles of neoadjuvant chemotherapy (comprising cyclophosphamide, doxorubicin, and vincristine), following which they were randomized to surgical or non-surgical groups. The study found no disparity in OS between the two groups, with both displaying a 2-year survival rate of 20%. The limitations of these earlier studies include the lack of chemotherapy in the MRC study and the absence of platinum-based chemotherapy in the neoadjuvant phase in the LCSG study. In recent years, however, advancements in diagnostic and surgical technologies, along with the emergence of novel therapeutic modalities like immunotherapy, have necessitated a reevaluation of the role of surgery in SCLC management. This has prompted extensive reconsideration in a series of large-scale retrospective observational studies conducted recently (17, 18).

In NSCLC patients, the results of the Checkmate 816 study showed a significant enhancement in the pCR rate with neoadjuvant nivolumab combined with chemotherapy as compared to neoadjuvant chemotherapy alone (24% vs. 2.2%, p< 0.0001), it also demonstrated a noticeably extended EFS (31.6 months vs. 20.8 months, p=0.0052), and the safety profiles of both treatment groups were analogous, exhibiting similar incidences of grade 3-4 treatment-related adverse events (TRAE) at 34% and 37%, respectively (7). Moreover, the study emphasized that neoadjuvant immunotherapy combined with chemotherapy, when compared to chemotherapy alone, does not impact the surgery rate, timing and completeness of resection, nor does it escalate the operational complexity or the risk of surgical complications. And for SCLC, several previous studies have corroborated that the combination of chemotherapy combined with immunotherapy significantly improves the overall response rate (ORR), PFS and OS of ES-SCLC compared to chemotherapy alone (9–11). Previous case reports and retrospective studies have also reported the role of neoadjuvant immunotherapy combined with chemotherapy in LS-SCLC. Liu et al. conducted a retrospective study and the results showed that neoadjuvant immunotherapy combined with chemotherapy followed by surgical resection is safe and effective in patients with stage I-IIIA SCLC (19). Next, Zhang et al. reported a case of more than 23 months of event-free survival after receiving neoadjuvant tislelizumab combined with chemotherapy (8). Later, a multicenter single-arm study demonstrated that neoadjuvant atezolizumab combined with chemotherapy significantly improved the pCR of resectable SCLC, and AEs were controllable (20). In addition, a network meta-analysis conducted by Wu et al. on the efficacy of first-line immune checkpoint inhibitors (ICIs) combined with chemotherapy revealed that, in comparison to other ICIs, the combination of serplulimab and chemotherapy was most likely to yield superior PFS and OS in patients with ES-SCLC (21). Our study suggests that the treatment strategy of neoadjuvant chemotherapy combined with serplulimab is also applicable to potentially resectable SCLC cases, with the ability to achieve postoperative pCR and ultimately provide patients with an extended EFS.

**Figure 4:**
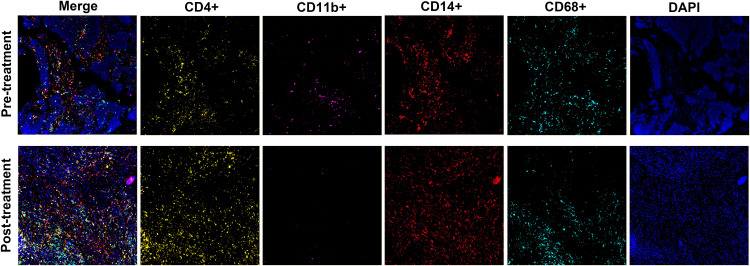
Tumor immune microenvironment before and after treatment.

To explore the changes in the tumor immune microenvironment before and after neoadjuvant immunochemotherapy, we performed multiplex immunofluorescence staining on T cells, macrophages, monocytes, and myeloid-derived suppressor cells (MDSC) in the patient’s preoperative and postoperative specimens (**Figure 4**). We found that the infiltration of T cells and monocytes in patients’ postoperative specimens increased significantly, while MDSC and macrophages decreased significantly. In recent years, different immune cell subpopulations have been divided into “positive” immune cell subpopulations and “negative” immune cell subpopulations. “Positive” immune cell subpopulations include CD4+T cells, NK cells CD8+T cells, etc. (22–24), while “negative” immune cell subpopulations include Tregs, tumor-associated macrophages, myeloid-derived suppressor cells (MDSCs), etc (25–27). Therefore, after treatment, the abundance of “positive” immune cell subpopulations increases and the abundance of “negative” immune cell subpopulations decreases, which may indicate that the patient will benefit from immunotherapy. As shown in the case we report.

However, it is important to highlight a discrepancy in the number of preoperative neoadjuvant therapy cycles between the Checkmate 816 study and our case. In the former, patients underwent three cycles of therapy, while in our case, the patient received five cycles of neoadjuvant serplulimab combined with chemotherapy before surgery, aligning with the cycle of neoadjuvant therapy implemented in the LCSG study. As far as we know, most phase II/III clinical trials involving neoadjuvant immunotherapy, either as monotherapy or in combination with chemotherapy, employ 2-4 cycles (7, 28–31). Nonetheless, due to the absence of direct comparisons among different cycle groups, a definitive conclusion regarding the optimal number of treatment cycles for neoadjuvant immunotherapy combined with chemotherapy remains elusive. To address this issue, He et al. conducted a retrospective study, which found that the major pathologic response (MPR) rates of NSCLC patients who received 2, 3, 4, and ≥ 4 cycles of neoadjuvant immunotherapy combined with chemotherapy were 44.8%, 61.4%, 66.7%, and 40.0%, respectively (32). To adjust for the potential of subjectively reducing treatment cycles due to significant tumor downstaging, they performed a subgroup analysis on patients who achieved complete or partial response (CR/PR) and found that MPR rates of 2, 3, 4, and ≥ 4 cycles are 43.8%, 71.0%, 71.4%, and 33.3%, respectively. In summary, extending NSCLC neoadjuvant immunotherapy combined with chemotherapy to 3-4 cycles has shown a higher MPR rate than 2 cycles, and this extension to 3-4 cycles remains beneficial even if imaging reveals CR/PR. In our study, the rationale behind administering five cycles of preoperative neoadjuvant therapy hinged on several factors. First, the high risk of distant metastasis in SCLC suggests that additional treatment cycles could potentially eradicate micrometastases. Second, the size of the primary tumor and lymph node size continued to shrink before the fifth cycle of treatment. After the fifth cycle, the tumor size stabilized, meeting the conditions for surgical R0 resection. And before the fifth cycle, NSE continued to decline, and after the fifth cycle, the NSE was in a stable period. Moreover, we observed no TRAE of any grade, attesting to the safety and feasibility of this treatment strategy.

Limitations and challenges: First, although the probability of tumor progression in SCLC during the neoadjuvant treatment phase is low, if disease progression occurs, patients may lose the opportunity for local treatment; Second, clinicians generally use imaging to evaluate the response of solid tumors to systemic therapy, but the Response Evaluation Criteria in Solid Tumors (RECIST) cannot accurately evaluate ICI neoadjuvant therapy; Third, the patient’s postoperative pathology achieved pCR, suggesting that the patient has the potential to be cured. The relationship between pCR after neoadjuvant therapy and the prognosis of NSCLC has been confirmed by many studies, but whether pCR after neoadjuvant therapy for SCLC is related to prognosis (DFS/OS) has a close correlation, which has yet to be proven by research, and we still need to remain cautiously optimistic; Fourth, it is still questionable whether sequential surgery after neoadjuvant treatment can challenge the existing standard treatment of concurrent chemoradiotherapy, which is also the basis for our plan for subsequent clinical trials; Fifth, after neoadjuvant treatment followed by surgery, there is still a lack of standard reference for implementation decisions of adjuvant chemotherapy, adjuvant radiotherapy, adjuvant immunotherapy and even prophylactic cranial radiotherapy. These may affect prognosis and should be designed and explored as scientifically as possible in future studies.

## Conclusions

Our case illustrates that neoadjuvant serplulimab combined with chemotherapy followed by surgery could potentially serve as an effective therapeutic strategy for the curative treatment of locally advanced SCLC. Future prospective randomized controlled studies are needed to further validate our findings.

## Data availability statement

The raw data supporting the conclusions of this article will be made available by the authors, without undue reservation.

## Ethics statement

The studies involving humans were approved by the ethics review committee of West China Hospital Sichuan University. The studies were conducted in accordance with the local legislation and institutional requirements. Written informed consent for participation was not required from the participants or the participants’ legal guardians/next of kin in accordance with the national legislation and institutional requirements. Written informed consent was obtained from the individual(s) for the publication of any potentially identifiable images or data included in this article.

## Author contributions

TM: Conceptualization, Formal analysis, Methodology, Writing – original draft, Writing – review & editing. TW: Funding acquisition, Supervision, Writing – review & editing. CFL: Data curation, Writing – review & editing. DJ: Data curation, Writing – review & editing. QHZ: Conceptualization, Supervision, Writing – review & editing.
